# Partnership development of the COVID-19 Front Door: a best evidence resource

**DOI:** 10.5195/jmla.2021.1353

**Published:** 2021-10-01

**Authors:** Nancy J. Allee, Charles P. Friedman, Allen J. Flynn, Chase Masters, Kai Donovan, Jane Ferraro, Roma Patel, Joshua C. Rubin

**Affiliations:** 1 nallee@umich.edu, Director, Taubman Health Sciences Library & STEM, University Library, and Joint Faculty, Department of Learning Health Sciences, University of Michigan Medical School, Ann Arbor, Michigan; 2 cpfried@umich.edu, Josiah Macy Jr. Professor of Medical Education and Chair, Department of Learning Health Sciences, University of Michigan Medical School, Ann Arbor, Michigan; 3 ajflynn@umich.edu, Assistant Professor, Research Analyst, and Technology Lead, Department of Learning Health Sciences, University of Michigan Medical School, Ann Arbor, Michigan; 4 mastersc@umich.edu, Enabling Technologies Informationist and Technology and Strategic Project Manager, Taubman Health Sciences Library & STEM, University Library, University of Michigan, Ann Arbor, Michigan; 5 annepz@umich.edu, Media and Learning Technologies Specialist, Taubman Health Sciences Library & STEM, University Library, University of Michigan, Ann Arbor, Michigan; 6 jferraro@med.umich.edu, Clinical Health Project Manager, Department of Learning Health Sciences, University of Michigan Medical School, Ann Arbor, Michigan; 7 romap@umich.edu, Graduate Student, Master of Health Informatics Program, School of Information, University of Michigan, Ann Arbor, Michigan; 8 rubinjc@umich.edu, Program Officer, Learning Health System Initiatives, Department of Learning Health Sciences, University of Michigan Medical School, Ann Arbor, Michigan

## Abstract

This project describes the creation of a single searchable resource during the pandemic, called the COVID-19 Best Evidence Front Door, with a primary goal of providing direct access to high-quality meta-analyses, literature syntheses, and clinical guidelines from a variety of trusted sources. The Front Door makes relevant evidence findable and accessible with a single search to aggregated evidence-based resources, optimizing time, discovery, and improved access to quality scientific evidence while reducing the burden of frontline health care providers and other knowledge-seekers in needing to separately identify, locate, and explore multiple websites.

Early on in the COVID-19 pandemic, a need for evidence-based information was recognized, and as a partnership endeavor, the Taubman Health Sciences Library and the Department of Learning Health Sciences at the University of Michigan worked together to create a searchable database of relevant resources for frontline health care professionals. A team of informationists, faculty, researchers, and administrators developed a rapid prototype resource, and then a group of volunteers collaborated with the team to identify and review documents for inclusion.

This effort resulted in the creation of a single searchable resource, called the COVID-19 Best Evidence Front Door, providing direct access to high-quality meta-analyses, literature syntheses, and clinical guidelines from a variety of trusted sources. The Front Door makes relevant evidence findable and accessible with a single search, reducing the search time for frontline health care providers and other knowledge-seekers in locating high-quality, evidence-based COVID-19 information resources (Figure 1). The database also identifies and organizes guidelines from a variety of reputable sources to benefit clinical leadership in more readily developing local policies and procedures for preventing, monitoring, diagnosing, and treating COVID-19.

**Figure 1:**
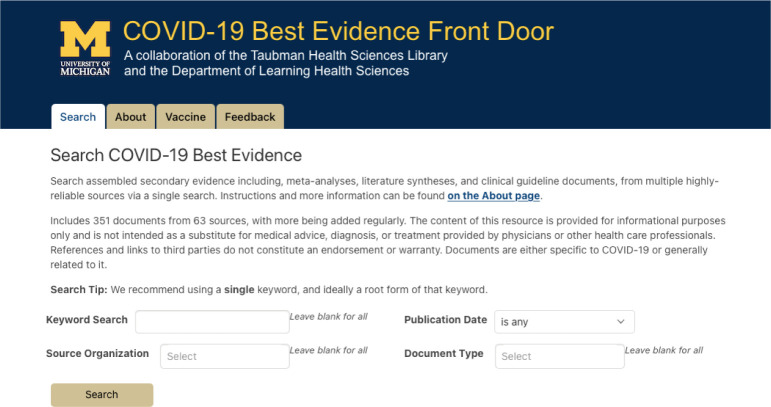
COVID-19 Best Evidence Front Door web portal

To determine which documents to include in the database, based on the best available evidence, a draft set of criteria was developed and then rapid pilot tested by five project team members who applied the criteria to guideline searches [1]. Following the initial pilot testing, document searches were expanded, using more targeted approaches, such as searching for guidelines about drug treatments for COVID-19. The criteria were then refined to arrive at the set of six inclusion criteria (Figure 2).

**Figure 2:**
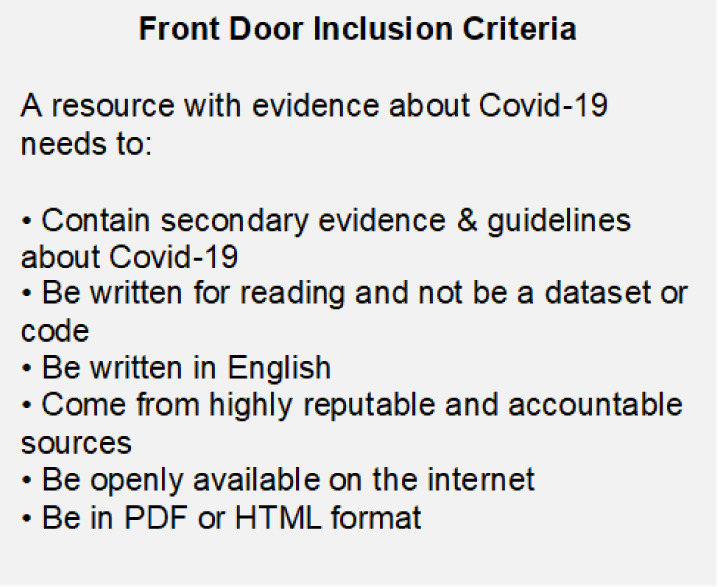
Front Door inclusion criteria

To organize documents for review and to manage the workflow, two online Google spreadsheets were created and maintained as the “document base.” The first spreadsheet is fluid and ever-changing and is used by project team members, supplemented by a group of interested volunteers, as a working list of potential articles for review with a core set of information provided about each guideline, including complete title, dates of publication, working hyperlink, and keywords. The second spreadsheet is used to capture and manage static, moment-in-time snapshots of the document base. These snapshots are accessible and searchable online.

The Front Door database has been developed using Knack and Vee Technologies. Knack is a platform for creating a combined website and searchable database without requiring a lot of coding expertise. The interface is easy to use and intuitive, making data quickly and securely searchable and sharable online. Librarians and other information providers can use Knack as a customizable tool to expedite the creation of a website that is more than just descriptive text and hyperlinks to other resources because it also enables the organizing, structuring, and searching of data from existing spreadsheets.

Updating the database becomes an efficient process as well. The Front Door prototype website and database were created and ready for user testing within an eight-day period, and once developed, the updating of the database is now a six-step process, taking approximately thirty minutes of time. The database accepts CSV file uploads, which enables easy integration into the Front Door project team's Google Sheets workflow and uploading of document base snapshots. A multipart search capability was also deployed, allowing users to search the document base by keyword, publication date, source organization, and document type. Knack provided their fee-based services and software free of charge for ninety days because of the primary focus on providing access to COVID-19 resources during the pandemic. Since the building and testing of the resource was a concentrated effort, requiring only slightly more than a week of time, the Front Door's availability was able to be announced in April 2020 within a month of COVID-19 surging and a concerted move to socially distanced and remote work.

A similar partnership with Vee Technologies enhanced the ability to more rapidly keep up with fast-changing content about COVID-19. Vee Technologies is a company that provides bots that automatically search the web for information on specified resources. This automation is an efficiency tool, enabling quicker identification of resources of interest and improving data collection. For the Front Door project, this technology featured an automation bot to scrape COVID-19 documents from selected source organizations' websites. The company sent emails, twice per week, with updated lists of new COVID-19 document metadata from the selected websites for review for possible inclusion in the Front Door. This service was provided free of charge, with only a request for acknowledgment on the Front Door website, and it enabled quicker identification of potential COVID-19 documents for review and inclusion.

The Front Door is publicly available at <http://covid19bestevidence.org/>. The site currently provides searchable access to over 350 documents from over 60 different sources (as of June 2, 2021). Google Analytics was added to the Front Door prior to launch to capture access and usage data. The database has been utilized by over 3,400 visitors to the site between April 21, 2020, and May 27, 2021, with a total of 4,383 sessions (“the period of time a user is actively engaged with [the] website”) [2]. To date, the majority of users, over 74%, are based in the US, followed by the UK, Canada, and Germany.

New features continue to be explored, including interactive visual document filtering to further increase ease-of-use and user engagement. The interactive pie charts present users with a method for grouping and filtering documents based on document criteria and search terms of interest (Figure 3). Once the user has created their own pie chart, they can click on any slice in the chart to see the corresponding documents in a table (Figure 4).

**Figure 3:**
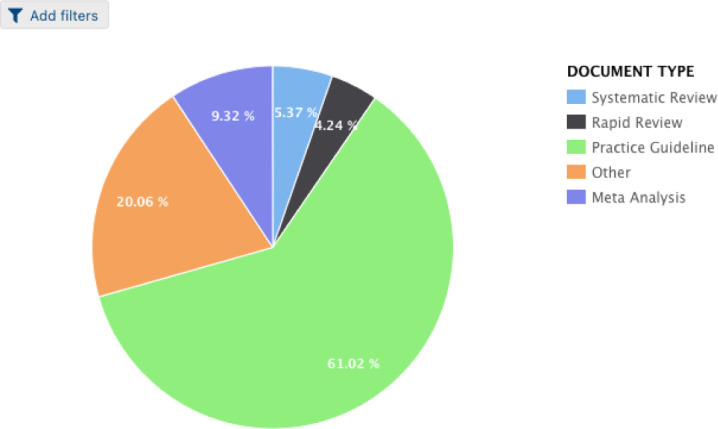
Interactive visual document filtering chart

**Figure 4:**
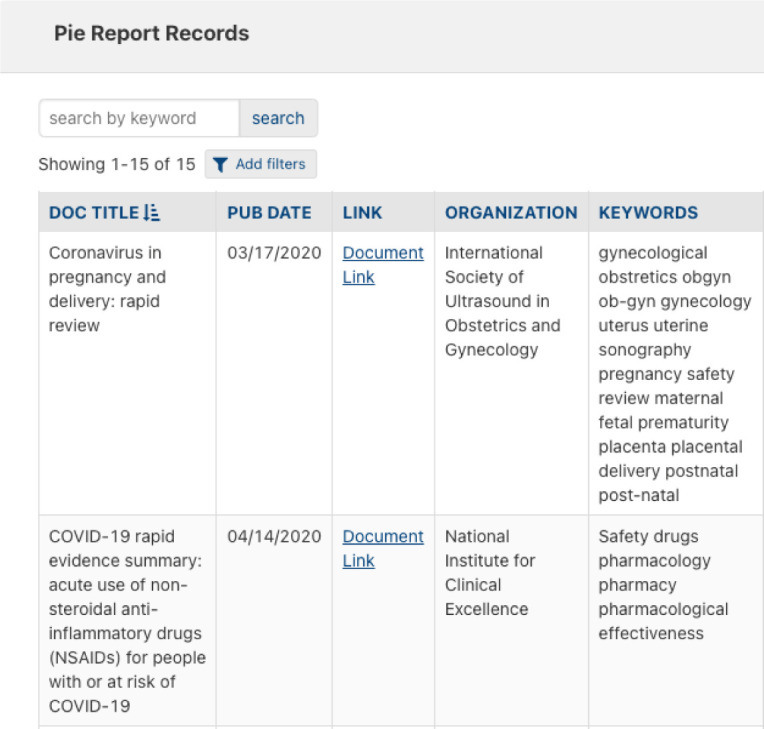
Interactive record display

Content continues to be updated as well, including documents on recent vaccine-related evidence as they become available. To increase ease of accessibility for this information, a “Vaccine” tab has been added, which includes links to vaccine articles generated by four PubMed queries. Two of the four queries search title and title/abstract of FDA-authorized COVID-19 vaccine information, and the remaining two queries search title and title/abstract of COVID-19 vaccine information in general.

Visitors to the site can also sign up for weekly updates which include new evidence documents as well as preexisting documents that have been revised.
